# Strengthening advocacy and policy change for infant and young child feeding

**DOI:** 10.1111/mcn.12749

**Published:** 2019-02-22

**Authors:** Isabelle Michaud‐Létourneau, Marion Gayard, David Louis Pelletier

**Affiliations:** ^1^ Department of Social and Preventive Medicine, School of Public Health Université de Montréal Montreal Quebec Canada; ^2^ Department of Family Medicine and Emergency Medicine Université de Sherbrooke Longueuil Quebec Canada; ^3^ Division of Nutritional Sciences Cornell University Ithaca New York USA

**Keywords:** advocacy, code of marketing of breast‐milk substitutes, contribution analysis, developmental evaluation, infant and young child feeding, policy change

## Abstract

The creation of environments that are more supportive of optimal infant and young child feeding (IYCF) requires countries to enact policies, such as those related to the Maternity Protection Convention, the International Code of Marketing of Breast‐milk Substitutes (the Code), and the Baby‐Friendly Hospital Initiative. However, challenges are experienced in the translation of international policy standards into national legal measures, and there is an important gap in understanding how countries achieve progress. Policy advocacy is a nearly universal feature, but there are methodological challenges and few studies evaluating strategies and effects. The purpose of this supplement to *Maternal & Child Nutrition* is to address those gaps. This supplement contains three papers that present findings from a real‐time evaluation of the advocacy efforts of Alive & Thrive (A&T), United Nations International Children's Emergency Fund (UNICEF), and partners, that sought to support governments in fostering enabling environment for optimal IYCF in Southeast Asia (SEA) and Africa. A combination of two emergent, theory‐based evaluation approaches was used: developmental evaluation and contribution analysis. The overall objective of the evaluation was to document the extent to which policy objectives were or were not achieved in each country and to identify the key drivers of policy change. One contribution of the supplement is a distinction between and illustration of triggers and drivers of policy change. Three main drivers of policy change were identified: (a) the use of an explicit advocacy approach; (b) the creation of a strategic group of actors; and (c) the realization of 15 critical tasks (more specifically for the Code). Each of the critical tasks has been identified as having triggered progress on the Code in those countries. This supplement provides evidence that the advocacy efforts of A&T, UNICEF, and partners contributed to enhanced IYCF policies in SEA and reveals how it helped to achieve progress. The insights contained in this supplement can serve as a guide for policy advocates for enhanced IYCF policies. A short communication puts findings into perspective within global context.

**Key messages**

- Fostering optimal environments for infant and young child feeding (IYCF) requires countries to enact policies, but challenges are experienced in the translation of international policy standards into national legal measures.
- Advocacy initiatives are an essential component, but the evaluation of their effects on policy change is rare and difficult.
- This real‐time evaluation based on contribution analysis and developmental evaluation identified three key drivers and 15 triggers for progress on the Code.
- The advocacy efforts of A&T, UNICEF, and partners in nine countries contributed to enhance IYCF policies.

## 1 INTRODUCTION

Creating enabling environments for infant and young child feeding (IYCF) remains a major challenge globally (Rollins et al., 2016). Enacting relevant policies is one step in creating environments that are more supportive of optimal breastfeeding and complementary feeding practices. There are several examples of policies that can help promote, protect, and support breastfeeding, as stipulated in the Innocenti Declaration of 1990 (UNICEF, 1990). The Maternity Protection Convention (No. 183) from the International Labour Organization is an instrument that provides for a minimum of 14 weeks of maternity benefit to women by ensuring sufficient time for birth, recovering, and nursing their children (International Labour Office, 2000). The *International Code of Marketing of Breast‐Milk Substitutes* (the Code) adopted by the World Health Assembly (WHA) in 1981, and updated regularly through subsequent WHA resolutions (World Health Organization [WHO], 2018a), represents the international policy framework for protecting breastfeeding against inappropriate marketing practices that negatively impact on breastfeeding. Another example is the Baby‐friendly Hospital Initiative (BFHI) that was launched by the WHO and the United Nations International Children's Emergency Fund (UNICEF) in 1991 and recently updated following a comprehensive consultation process to address some key challenges identified in BFHI implementation and to provide a more robust program (World Health Organization, 2018b). The original BFHI required compliance with the 10 steps for successful breastfeeding in addition to full compliance with the Code, which represented a separate condition (World Health Organization & UNICEF, 1989). In the updated guidelines, full application of the Code has been incorporated into Step 1 on infant feeding policies to better reflect its importance and inclusion in the initiative.

These international policy standards and frameworks are important norms and instruments, but countries experience challenges in translating them into their own legislation and in their enforcement. There is limited evidence and understanding in how countries achieve progress. Policy advocacy is clearly involved, but there are methodological challenges in evaluating the strategies and effects of advocacy (Gardner & Brindis, 2017; Glass, 2017) and few empirical studies in nutrition. The purpose of this supplement to *Maternal & Child Nutrition* is to address those gaps.

This supplement contains three papers that present findings from an evaluation of the advocacy efforts of Alive & Thrive (A&T), UNICEF, and partners, that sought to support governments in fostering enabling environment for optimal IYCF in nine countries in Southeast Asia (SEA) and Africa. Each paper examines distinct aspects of the advocacy efforts by multiple organizations working together to achieve a common goal. The first paper presents two of the key drivers of policy change and details the progress on the Code in the nine countries. The second paper shows how this progress was achieved in seven SEA countries, using the advocacy approach (third driver). The third paper examines issues related to the governance of large policy change initiatives using the collective impact framework. The supplement ends with a short communication from A&T and UNICEF that relate the findings of this supplement to latest development in global initiatives.

A second phase of advocacy efforts led by A&T–UNICEF began in 2014, with the aim of sharing the successful experience in Vietnam with six countries in SEA (Cambodia, Indonesia, Lao People's Democratic Republic, Myanmar, Thailand, and Timor‐Leste) and two African countries (Burkina Faso and Ethiopia) (Hajeebhoy et al., 2013). These advocacy efforts were based on the main strategies of the first phase of A&T involving a four‐part process for policy change: (a) establish and sustain partnership; (b) develop an evidence base; (c) develop messages and materials; and (d) build consensus (Alive & Thrive, 2016).

A real‐time evaluation of the advocacy efforts in those countries took place between 2015 and 2017. It drew upon recent advances in the field of evaluation to address the problem of attribution, which is a challenge in the context of advocacy and policy change evaluation because of the many and complex influences and interactions in these processes. The overall objective of the evaluation was to document the extent to which policy objectives were or were not achieved in each country and to identify the key drivers of policy change. A combination of two theory‐based evaluation approaches was used: developmental evaluation (Patton, McKegg, & Wehipeihana, 2015) and contribution analysis (Mayne, 2012). These methodologies allowed (a) engaging directly with policy advocates to document the experiences in the nine countries; (b) making credible claims about whether and how some advocacy activities carried out in those countries contributed to policy change; and (c) drawing some novel insights about the process.

The first paper of this supplement, “Translating the international code of marketing of breast‐milk substitutes into national measures in nine countries” presents two of the drivers of policy change (Michaud‐Létourneau, Gayard, & Pelletier, 2019a). One driver was the creation of a strategic group, which engaged key relevant actors and supported the government in carrying out 15 critical tasks throughout the policy cycle. This complete set of critical tasks represented the second driver. Each of the critical tasks has been identified as having triggered progress on the Code in some countries. Each of the critical task has also been linked to the different stages of the policy cycle that include agenda setting, development of the Code, adoption, preparation for implementation, monitoring and enforcement, and evaluation, learning, and adaptation. The paper illustrates how the strategic groups of actors in the participating countries have supported the governments in undertaking those tasks and how it influenced different stages of the policy cycle. The insights contained in this paper can serve as a guide for policy advocates who work on the Code in anticipating challenges at subsequent stages and develop more effective strategies. This is particularly important considering that the industry uses recurrently inappropriate marketing practices to reach more mothers, which negatively impacts breastfeeding. In addition, this paper describes the progress achieved in each of those countries during the evaluation period, based on major and intermediate accomplishments.

The second paper is entitled “Contribution of the Alive & Thrive‐UNICEF advocacy efforts to improve infant and young child feeding policies in Southeast Asia” (Michaud‐Létourneau, Gayard, & Pelletier, 2019b). This paper illustrates the application of contribution analysis to evaluate a policy advocacy initiative, which is scarce in the literature and, to our knowledge, non‐existent in nutrition. The contribution analysis was based on multiple sources of data collected through the application of developmental evaluation, which relies upon repeated interaction and feedback with and from the actors involved in the advocacy efforts. The paper describes how the six steps of contribution analysis were carried out: (a) clarify the cause–effect issue to be addressed; (b–c) develop the theory of change and gather existing evidence; (d) assemble and assess the contribution story; (e) seek out additional evidence; and (f) revise and strengthen the contribution story (Mayne, 2008; Van Melle et al., 2017). In addition to illustrating the methodology, the paper provides evidence that the advocacy efforts of A&T, UNICEF, and partners contributed to enhanced IYCF policies in SEA and reveals how it did so. This evidence identified the systematic use of the advocacy approach as the third key driver of policy change. Thus, this second paper demonstrates the value of contribution analysis to evaluate an advocacy initiative and adds to our understanding of the ways by which advocacy contributes to progress in policy change.

The third paper, “Enhancing governance and strengthening advocacy for policy change of large Collective Impact initiatives”, helped examine the governance of large initiatives seeking to achieve social or policy change (Michaud‐Létourneau, Gayard, & Pelletier, 2019c). The collective impact lens is applied to the governance of the A&T–UNICEF advocacy efforts through its five conditions: (a) backbone support (staff dedicated to the initiative); (b) common agenda; (c) mutually reinforcing activities; (d) continuous communication; and (e) shared measurement. This application brings two types of insights. First, it reveals some challenges, adaptations, and key elements needed when expanding a collective impact intiative operating at one level (e.g., one country) into one operating at multiple levels (e.g., multiple countries). Second, it provides guidance on how collective impact initiatives can add and strengthen policy advocacy, including the need to overcome the fear of advocacy, build advocacy capacity, and promote an understanding of all phases of the policy cycle.

Finally, a short communication links the findings from this evaluation to latest development in global initiatives (Bégin et al., 2019). The paper describes two instruments developed to help countries in advocating for better IYCF policies, a scorecard and an investment case, and it illustrates their use in Nigeria and China. Updates on global and regional platforms to improve IYCF policies, strategies and financing are also presented.

In summary, this real‐time evaluation undertaken in nine countries allowed the identification of three key drivers of policy change: (a) the use of an explicit advocacy approach; (b) the creation of a strategic group of actors; and (c) the realization of 15 critical tasks. These key drivers were instrumental in achieving significant progress. Whereas these substantive findings, and the evaluation methodologies applied in this study, relate to efforts to strengthen the Code, they also can provide guidance on other policy issues in nutrition and beyond.

## ETHICS APPROVAL

Ethics committees at both the University of Sherbrooke and FHI360 approved the research protocol.

## CONFLICTS OF INTEREST

The authors declare that they have no conflicts of interest.

## CONTRIBUTIONS

IML conceptualized this real‐time evaluation, played a leadership role at all stages, and articulated the idea of this supplement with DLP providing advice throughout. IML drafted the different sections of this specific manuscript. All authors read, commented, and approved the final manuscript.

## FUNDING

Alive & Thrive is funded by the Bill & Melinda Gates Foundation, the governments of Canada and Ireland and is managed by FHI 360.

## References

[mcn12749-bib-0001] Alive & Thrive (2016). A guide for public health advocacy: Tools and lessons learned from successful infant and young child feeding advocacy in Southeast Asia. http://www.aliveandthrive.org/wp‐content/uploads/2018/02/Guide_Infant_Child_Feeding_Advocacy.pdf

[mcn12749-bib-1012] Bégin, F. , Lapping, K. , Clark, D. , Taqi, I , Rudert, C. , Mathisen, R. , & Stathopoulos, Z. (2019). Real‐time evaluation can inform global and regional efforts to improve breastfeeding policies and programmes. Maternal & Child Nutrition, 15(Suppl. 2), e12774 10.1111/mcn.12774 30793544 PMC6519217

[mcn12749-bib-0002] Gardner, A. , & Brindis, C. (2017). Advocacy and policy change evaluation: Theory and practice. Stanford University Press.

[mcn12749-bib-0003] Glass, J. (2017). "Advocates change the world; evaluation can help": A literature review and key insights from the practice of advocacy evaluation. Canadian Journal of Program Evaluation, 32(1). 10.3138/cjpe.31039

[mcn12749-bib-0004] Hajeebhoy, N. , Rigsby, A. , McColl, A. , Sanghvi, T. , Abrha, T. H. , Godana, A. , … Sather, M. (2013). Developing evidence‐based advocacy and policy change strategies to protect, promote, and support infant and young child feeding. Food & Nutrition Bulletin, 34(Supplement 2), 181S–194S. 10.1177/15648265130343S205 24261076

[mcn12749-bib-0005] International Labour Office (2000). Maternity protection convention, 2000 (Revised) (ILO No. 183). *Annual review of population law* from https://http://www.ilo.org/dyn/normlex/en/f?p=NORMLEXPUB:12100:0::NO::P12100_ILO_CODE:C183 12344555

[mcn12749-bib-0006] Mayne, J . (2008). Contribution analysis: An approach to exploring cause and effect. Retrieved from https://cgspace.cgiar.org/bitstream/handle/10568/70124/ILAC_Brief16_Contribution_Analysis.pdf?sequence=1&isAllowed=y

[mcn12749-bib-0007] Mayne, J. (2012). Contribution analysis: Coming of age? Evaluation, 18(3), 270–280. 10.1177/1356389012451663

[mcn12749-bib-0010] Michaud‐Létourneau, I. , Gayard, M. , & Pelletier, D. L . (2019a). Translating the international code of marketing of breast‐milk substitutes into national measures in nine countries. Maternal & Child Nutrition, 15(Suppl. 2), e12730. 10.1111/mcn.12730 PMC6519018 30793543

[mcn12749-bib-0008] Michaud‐Létourneau, I. , Gayard, M. , & Pelletier, D. L. (2019b). Contribution of the Alive & Thrive‐UNICEF advocacy efforts to improve infant and young child feeding policies in Southeast Asia. Maternal & Child Nutrition, 15(Suppl. 2), e12683. 10.1111/mcn.12683 PMC6519196 30793546

[mcn12749-bib-0009] Michaud‐Létourneau, I. , Gayard, M. , Mathisen, R. , Phan, L. T. H. , Weissman, A. , & Pelletier, D. L . (2019c). Enhancing governance and strengthening advocacy for policy change of large Collective Impact initiatives. Maternal & Child Nutrition, 15(Suppl. 2), e12728. 10.1111/mcn.12728 PMC6519038 30793547

[mcn12749-bib-0011] Patton, M. Q. , McKegg, K. , & Wehipeihana, N. (2015). Developmental evaluation exemplars: Principles in practice. Guilford Publications.

[mcn12749-bib-0012] Rollins, N. C. , Bhandari, N. , Hajeebhoy, N. , Horton, S. , Lutter, C. K. , Martines, J. C. , … Group, The Lancet Breastfeeding Series (2016). Why invest, and what it will take to improve breastfeeding practices? The Lancet, 387(10017), 491–504. 10.1016/S0140-6736(15)01044-2 26869576

[mcn12749-bib-0013] UNICEF (1990). The Innocenti declaration on the protection, promotion and support breastfeeding. Italy: Florence.

[mcn12749-bib-0014] Van Melle, E. , Gruppen, L. , Holmboe, E. S. , Flynn, L. , Oandasan, I. , & Frank, J. R. (2017). Using contribution analysis to evaluate competency‐based medical education programs: It's all about rigor in thinking. Academic Medicine, 92(6), 752–758. 10.1097/ACM.0000000000001479 28557934

[mcn12749-bib-0015] World Health Organization (2018a). Code and subsequent resolutions. October 2018, Retrieved from http://www.who.int/nutrition/netcode/resolutions/en/

[mcn12749-bib-1000] World Health Organization (2018b). Implementation guidance: Protecting, promoting and supporting breastfeeding in facilities providing maternity and newborn services: The revised baby‐friendly hospital initiative. 29565522

[mcn12749-bib-0016] World Health Organization, & UNICEF (1989). Protecting, promoting and supporting breast‐feeding: The special role of maternity services.

